# Research progress on the biological modifications of implant materials in 3D printed intervertebral fusion cages

**DOI:** 10.1007/s10856-021-06609-4

**Published:** 2021-12-23

**Authors:** Shan Li, Yifan Huan, Bin Zhu, Haoxiang Chen, Ming Tang, Yiguo Yan, Cheng Wang, Zhihua Ouyang, Xuelin Li, Jingbo Xue, Wenjun Wang

**Affiliations:** 1 Department of Spine Surgery, The First Affiliated Hospital, Hengyang Medical School, University of South China, 69 Chuanshan Road, Hengyang, Hunan 421001 China; 2 Plastic and Cosmetic Surgery, Hunan Want Want Hospital, Changsha, China; 3 R&D Department, Hunan Yuanpin Cell Biotechnology Co. Ltd., Changsha, China

## Abstract

Anterior spine decompression and reconstruction with bone grafts and fusion is a routine spinal surgery. The intervertebral fusion cage can maintain intervertebral height and provide a bone graft window. Titanium fusion cages are the most widely used metal material in spinal clinical applications. However, there is a certain incidence of complications in clinical follow-ups, such as pseudoarticulation formation and implant displacement due to nonfusion of bone grafts in the cage. With the deepening research on metal materials, the properties of these materials have been developed from being biologically inert to having biological activity and biological functionalization, promoting adhesion, cell differentiation, and bone fusion. In addition, 3D printing, thin-film, active biological material, and 4D bioprinting technology are also being used in the biofunctionalization and intelligent advanced manufacturing processes of implant devices in the spine. This review focuses on the biofunctionalization of implant materials in 3D printed intervertebral fusion cages. The surface modifications of implant materials in metal endoscopy, material biocompatibility, and bioactive functionalizationare summarized. Furthermore, the prospects and challenges of the biofunctionalization of implant materials in spinal surgery are discussed.

**Figure:**
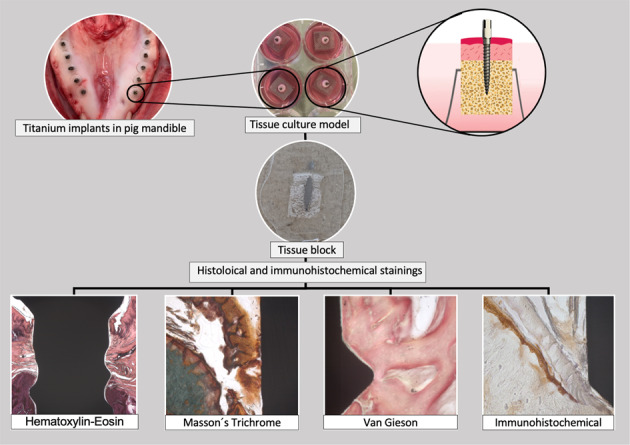
Fig.a.b.c.d.e.f.g As a pre-selected image for the cover, I really look forward to being selected. Special thanks to you for your comments.

Fig.a.b.c.d.e.f.g As a pre-selected image for the cover, I really look forward to being selected. Special thanks to you for your comments.

## Introduction

Spinal interbody fusion systems are widely used in the field of spinal surgery [1]. To accelerate osteogenic fusion after the insertion of spinal implants, three strategies have been reported, usually to improve the bone-implant interface morphology and promote bone-implant adhesion. The first strategy is to physically modify the material-bone interface by adjusting the surface roughness or porosity to increase the migration and proliferation of bone cells and ultimately promote the osseointegration of endophytes [2–7]. The second strategy is to chemically modify the surface of the endophytic material to enhance its biological activity, thereby stimulating bone regeneration and differentiation [8]. The third strategy is to modify the biological activity of the surface of the endophyte. As the endophyte material changes over time, it promotes cell recruitment, proliferation on the surface and in the surface, and finally differentiates into bone tissue to promote the biological functionalization of intervertebral fusion.

Bone regeneration is a complex coordinated physiological response of the body to bone defects [9–11] involving unknown signaling pathways and processes that are regulated by different cells, cytokines and growth factors [12–14]. Autologous bone transplantation remains the gold standard for bone regeneration treatment [15, 16]. Although autologous bone transplantation has obvious advantages, such as immunocompatibility, excellent bone conduction, osteoinduction and osteogenic properties, its applications are limited due to the insufficient supply of donor tissue and the risk of complications at the donor site. Similarly, the use of allografts and xenografts is limited by the immune response or infection [17–19]. The autologous bone grafts, allografts and xenografts in the above methods can significantly promote bone regeneration. However, the main limitation of bone substitutes is their lack of interactions that support the complex cell-osteogenic microenvironment [20]. Therefore, combining bone substitutes with cells to regulate osteogenic differentiation is an important direction in bone regenerative medicine.

## The development history of plant materials in the spine

In the middle of the 20th century, Cloward and others first proposed posterior lumbar interbody fusion, which is still one of the basic methods of spinal surgery [21]. In the 1980s, Bagby, Kuslich and others designed the first intervertebral fusion cage made of hollow stainless steel, which is suitable for the anatomical structure of the human body based on the compression and stabilization effects of expansion [21, 22]. In recent years, research on implant systems in the spine has intensified. Spine interbody fusion cages can not only store autologous bone particles but also restore the height between the vertebral bodies. These cages have become an ideal bone graft substitute material [23]. In order to avoid the overall stability of the spine, the loss of the lordosis angle, and the settlement or displacement of the internal plant after the operation, the spine interbody fusion cage is usually used in combination with a spine internal fixation system such as a pedicle screw internal fixation system. While effectively preventing the loss of the height between the vertebral bodies, the abundant local blood supply is obtained due to the expansion of the vertebral bodies to promote a higher rate of osteogenesis and fusion, and better maintain the advantages of the physiological curvature of the spine of the postoperative patient. Long-term follow-up shows that the spinal interbody fusion system has a better fusion rate and higher patient satisfaction [23].

In the development of spinal surgery, despite the rapid developments in biomaterials and tissue engineering, the development of spinal implants remains challenging due to the limitations of autogenous and allogeneic bone transplantation [19]. In addition to the application of support to patients who need osteotomies, such as those with severe angular kyphosis and spinal tumors, these limitations put forward higher requirements for matching spinal support performance, and the research and development of spine implants will become a new direction for bionic materials and personalized 3D printing customization.

## Application of 3D printing technology

### Using 3D printing technology to make spinal implants

3D printing technology is also known as Additive Manufacturing (AM), which is based on a digital model that integrates computer-aided manufacturing, numerical control technology, laser technology, polymer materials and three-dimensional computed tomography (CT) technology. 3D printing technology can quickly and accurately print the diseased area according to the patient’s personalized anatomical characteristics and clinical needs; thus, the required anatomical models and appropriate internal implants should be used clinically. Patients with spinal degenerative diseases often have variations in the anatomical parameters of the vertebral body, especially large changes in the height of the intervertebral space, and traditional mass production of spinal implants cannot fully meet clinical needs. Therefore, personalized customization of spinal implants has become necessary.

Currently, the main printing materials on the market are titanium and its alloys. Titanium has excellent corrosion resistance, good biocompatibility and good mechanical properties and is widely used in biomedical implants. However, the difference between Young’s modulus of Ti implants (110 GPa) and bone (10–30 GPa) results in a stress shielding effect after implantation in the body. 3D-printed customized porous titanium implant materials can adjust and optimize the parameters of the spinal implant, changing the porosity, connectivity and hole diameter by setting to finally effectively control the strength and elastic modulus of the stent and obtain the target porous titanium alloy spinal implant [24]. For implants with highly porous structures, there is greater surface contact at the bone-implant interface, and bone tissue and blood vessels can grow into the pores on the porous titanium [25], providing more space for bone integration and mechanical interlocking [26]. Studies have shown that the minimum pore size for significant bone growth should be between 75 and 100 µM [27] but the best-observed range was between 100 and 135 µM. However, to promote bone formation and angiogenesis, more than 300 µM of pores are needed [28]. 3D printing technology can independently set parameters, and Young’s modulus of the material can be adjusted to be similar to that of bone (average compressive modulus of elasticity 1.41 ± 0.007 GPa), which is between that of cortical bone and cancellous bone, making its clinical application adaptability increasingly higher [29, 30].

### 3D printing technology realizes material biofunctionalization

The ideal bone tissue engineering scaffold has good biocompatibility, bone tissue integration ability and suitable mechanical properties with an internal implant structure design that is consistent with the anatomical structure of natural bone tissue. 3D printing technology can measure the relevant anatomical spine parameters of patients with degenerative diseases, allowing the personalized custom anatomical spinal interbody fusion cage to have an improved fit on the bone surface, which can better solve the problem of traditional prostheses not matching the patient’s bones. Simplifying the operation steps also promotes a reduction in tissue damage. 3D printing of anatomical spinal implants has been gradually applied in clinical practice. With the development of AM technology, great progress has been made. For example, Professor Liu Zhongjun’s team from the Third Hospital of Peking University completed the world’s first 3D-printed artificial customized axial replacement for cervical malignant tumors [30, 31]. However, the lack of biological activity on the surface of the titanium alloy spinal implant is still an urgent problem in the osseointegration process [8]. The rapid recruitment of osteoblasts is a necessary prerequisite for the effective repair of bone defects. Some studies have shown that rough nanosurfaces can build a suitable microenvironment for cell growth. Therefore, the study of biomimetic implants constructed by increasing the surface roughness of materials to promote cell adhesion and osteogenic differentiation will receive increasing attention [30].

**Fig. 1:**
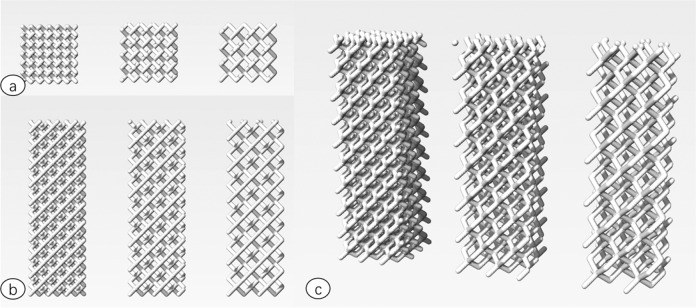
Three-dimensional model diagram of pore structure ((**a**) sagittal plane (**b**) coronal plane (**c**) 3D stereo view (1) diamond structure (2) octahedron structure (3) dodecahedron structure).

**Fig. 2:**
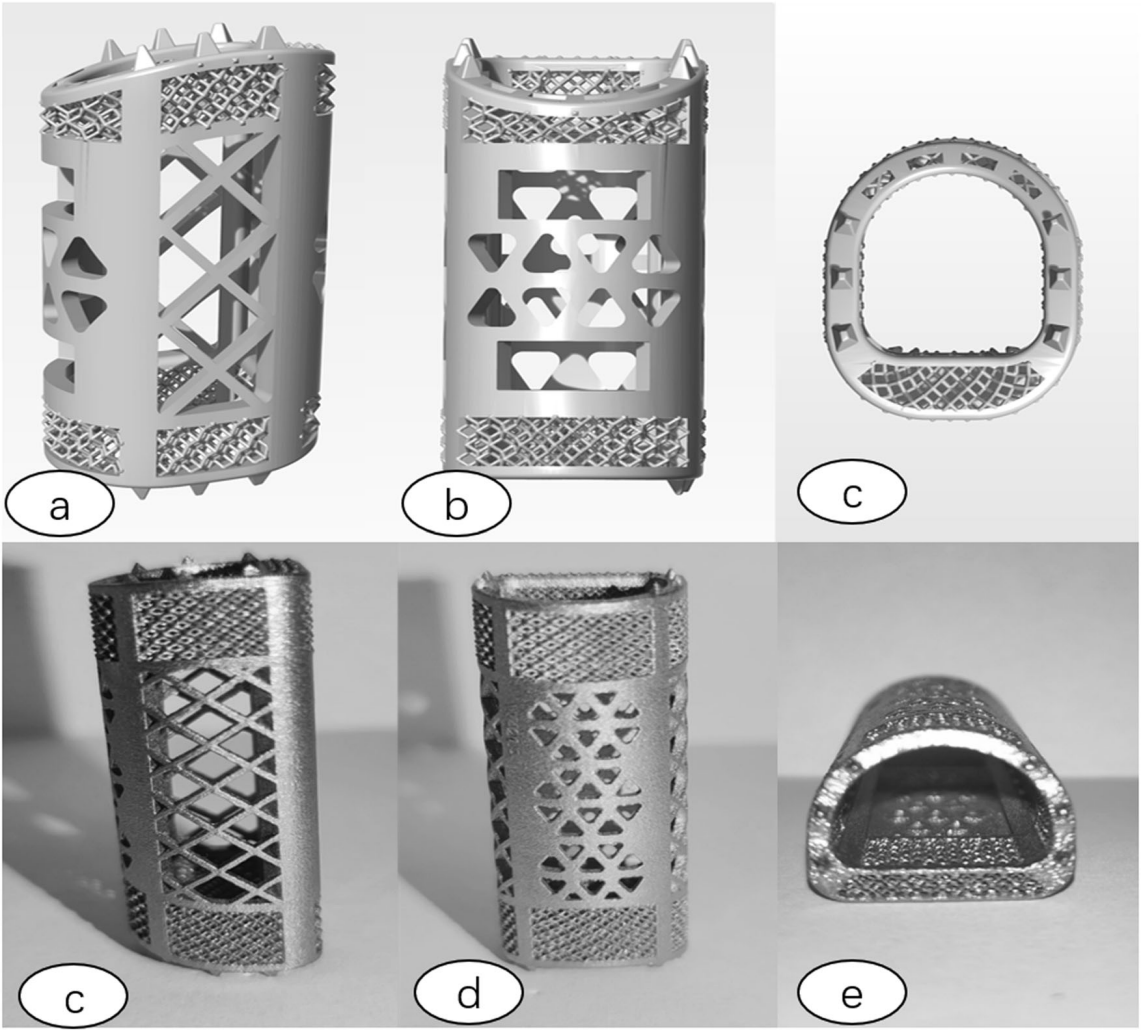
Bionic 3D printing porous titanium alloy bracket Schematic and physical drawings ((**a**) 3D three-dimensional side view (**b**) 3D three-dimensional front view (**c**) 3D three-dimensional top view (**d**) 3D three-dimensional side view (**e**) 3D three-dimensional front view (**f**) 3D three-dimensional top view).

This group independently designed a 3D printing porous titanium alloy bone substitute material by using synchrotron radiation imaging technology. The unit structure of material pore structure can design three kinds of spatial structures, including cylindrical rod diamond structure, cylindrical rod octahedron structure and cylindrical rod dodecahedron structure. The aperture range is 400–1500 μM. The range of rod diameter is 150, 200 and 250 μM (Fig. 1). The 3D data information of goat endplate microstructure was analyzed for modeling design, and the characteristics of natural cervical vertebral endplate such as pore size, porosity and pore connectivity were copied. By analyzing the 3D data of the microstructure of the goat vertebral body, the 3D printing modeling design is carried out, and a scaffold with the same micromechanical structure of the goat vertebral body endplate is produced. We call it the bionic printing anatomical scaffold material (Fig. 2). It is expected that the bionic 3D printing titanium alloy stent can maintain the spinal stability after bone graft fusion, enhance the effect of bone graft fusion, guide the bone growth in the pore structure, and effectively reduce the complications caused by the traditional titanium mesh sinking.

## Surface modification of plant materials in the spine

### Chemical and physical modification of endophyte materials

There have been different technical methods to improve the surface biological properties of such implants by coating or changing its surface morphology. The technology of mechanical, chemical or biomimetic modification of the surface of materials is changing with each passing day. Among these, biological functionalization of the material surface, including the immobilization of various peptide sequences, proteins, and even drug molecules, has been gradually developed [32, 33].

Although titanium is the most biocompatible metal material, it lacks biological activity and cannot induce bone regeneration or directly integrate with bone after implantation [8]. Therefore, the development of titanium-based biomaterials that can achieve biofunctionalization and ensure the ideal performance of the implants is a very important topic. Researchers have used micro-arc oxidation (MAO) technology to adjust the crystallinity and morphological characteristics to improve the biological properties of the titanium surface [34, 35]. The most common and effective way to improve the osseointegration of titanium and its alloys is to introduce a layer of hydroxyapatite (HA) coating to promote the transformation of the surface properties of the endophyte from a biologically inert surface to a biologically active surface [36, 37]. However, both the overall performance and biological activity of HA are not stable [38, 39]. Compared with pure HA, silicon-doped HA has higher dissolution and ion release rates in the body, which can provide a sufficient ion source for new bone formation. Therefore, implants with silicon-doped HA have can better promote angiogenesis and osteogenic regeneration [40, 41].

Biological functionalization of the inner implant surface can not only promote cell adhesion and proliferation but also trigger bacterial adhesion and the formation of bacterial biofilms and cause inflammation and infection, leading to implantation failure of the inner implant [42]. Some researchers fix antibacterial drugs on the surface of the inner implants to avoid spontaneous inflammatory reactions after the inner implants are implanted in the body. Commonly used methods of drug immobilization include physical adsorption [43], chemical grafting [44], polymer coatings containing antibacterial drugs [45], silver coatings [46], etc. However, these strategies also have drawbacks. For example, the adsorption method can cause the excessive release of antibiotics, causing cytotoxicity and increasing the risk of bacterial resistance [47]. Although chemical grafting avoids excessive drug release, the process of chemical covalent bonding may destroy the molecular structure of the drug and affect its biological functions. Polymer coatings with high hydrophobicity and an antibacterial agent is not conducive to the formation of strong biological bonds between the bone tissue and coating substrate. Although silver (Ag) has antibacterial properties, the release of Ag+ from silver-containing coatings will increase the cumulative concentration of Ag+ in the body, which will cause toxic side effects in body tissues [46]. With further study of the antibacterial properties of materials, biodegradable bovine serum albumin (BSA) nanoparticles (BNPs) have been used as carriers of many drugs [48–50]. As the carrier partner, BNPs have not only good biocompatibility and non immunogenicity but also high loading efficiency so that the antibacterial drugs attached to the surface of the inner implant can be implanted into the body for slow release [51]. Therefore, it is expected that antibiotic-loaded BNPs can maintain a stable blood concentration of antibiotics for a long period without affecting the biological functions of the substrate surface and cell physiological functions so that the drugs can exert better effects and enhance the antimicrobial activity of the endogenous implants.

Studies have shown that the structure of strontium can change from flakes to needle-shaped with increasing pH. In vitro experiments have shown that nanoneedle-like Sr (Sr: strontium) coatings prepared under high pH conditions can significantly inhibit the activity of osteoclasts, facilitate the adhesion, diffusion, proliferation and osteogenic differentiation of mesenchymal stem cells (MSCs), and promote the formation of new bone to enhance endophyte osseointegration. This provides new theoretical guidance for the design of implant surface coating in the spine and brings new hope for reducing the complications of endophytes after surgical treatment of clinical osteoporosis [52].

With continuous in-depth research on the bionics of implants in the spine, the construction of a bionic natural extracellular matrix microenvironment on the surface of internal implants has also attracted widespread attention [53]. Studies have shown that extracellular matrix (ECM) proteins with cell-binding domains can play an important role in regulating cell adhesion, proliferation and differentiation. The combination of ECM proteins and HA can completely mimic the functions of inorganic and organic components in the natural bone matrix, becoming an ideal osteoconductive and osteoinductive functional coating [54]. This system not only gives physical support but also forms a natural bone matrix microenvironment to induce tissue regeneration [55]. Fabrizio et al. used a porous titanium metal scaffold made by 3D printing and gelatin cross-linking to the extracellular matrix of platelets that can effectively release low-dose growth factors after implantation in vivo. This scaffold is conducive to the growth of bone tissue and has shown good biocompatibility [56]. The creation of bionic titanium scaffolds provides a promising structural and functional dual bionic strategy for the design of metal implants with osteoinductive, antibacterial, bone regenerative and infection-preventing functions.

### Combined application of biology and biomaterials

In recent years, tissue engineering technology has combined biomedical engineering, cell biology and biomaterial science to provide new strategies for the treatment of bone defects [57–59]. To design scaffolds to induce bone formation and angiogenesis, biodegradable materials are made into porous shapes and combined with growth factors, drugs, genes or stem cells with different bioactive functions to promote cell material surface interactions, which play an important role in bone integration after spinal implant implantation. Tissue engineering guides the fate of cell transformation through growth factors and regulates the expression of functional genes related to bone marrow mesenchymal stem cell (BMSC) bone formation on biomaterial scaffolds, which has become a very promising method for the treatment of bone defects [60]. Currently, DNA plasmids [61, 62], short interfering RNA (siRNA) [63], microRNA (miRNA) [64], stem cell exosomes [65] and other functional gene regulatory molecules have been experimentally applied in bone tissue engineering. Plasmid DNA (pDNA) requires DNA to undergo transfection, transcription, translation and other processes to express the effector proteins [66]. Noncoding RNA only needs to enter the cytoplasm to regulate genes at the expression level, and studies have shown that this is related to the fine regulation of bone regeneration [64, 67–71]. Some researchers have found that miR-19b-3p can inhibit the expression of the negative osteogenic regulator SmurF1 [72]. The lentivirus pLVTHM-miR-19b-3p was used to transfect BMSCs to promote their osteogenic differentiation by inhibiting the expression of Smurf1. This provides a new strategy for bone tissue engineering using microRNA gene-modified BMSCs combined with poly(lactic acid) (PLLA)/polyhedral oligomeric silsesquioxane (POSS) scaffolds [73].

Hydrogels, as carriers of cells and biologically active molecules, are used in ECM bionic scaffolds to mediate cell and tissue regeneration [74, 75]. Developing a new generation of hydrogel-inorganic particle stem cell composite implant materials improves the physical and biological properties of engineered bone and cartilage tissue [76]. Genetically modified potato virus X (PVX) nanoparticles can produce bone-related functional peptides, which are ideal materials for preparing hydrogel nanocomposites in bone tissue engineering [77].

By adjusting the physical and chemical properties of the substrate to imitate the ECM of natural tissues, cell-substrate contact can be enhanced to regulate cell behavior. Recombination occurs under the influence of pressure that is equivalent to the cells passing into the environment [78]. Studies have shown that liquid crystal substrates improve surface biological activity to enhance cell affinity and osteogenic differentiation [79]. Using the adhesive polydopamine (PDOPA) as the reaction platform, chitosan oligosaccharide (COS) can be immobilized on the hydroxypropyl cellulose ester (OPC) substrate to generate an active OPC-PDOPA-COS liquid crystal substrate to simulate the ECM environment in vivo, enhancing the positive cell-substrate interaction to provide good support. The results showed that after inoculation of rat bone marrow mesenchymal stem cells (rBMSCs) with OPC-PDOPA-COSs, alkaline phosphatase (ALP) activity increased, calcium deposition increased, and bone-related gene (BMP-2, RUNx-2, COL-I and OCN) expression was upregulated. The viscoelasticity and deformability of the liquid crystal materials led to an active substrate, which will have broad application prospects as an engineering interface with living cells [80].

Bone morphogenetic proteins (bone morphogenetic proteins 2 and 7; BMP-2 and BMP-7), as growth factors, cytokines and metabolites, participate in the osteogenic differentiation of BMSCs [81] and play a key role in the regulation of bone induction and repair [82]. Traditional BMP-2 carriers include collagen, hyaluronic acid, polyethylene glycol diacrylate and gelatin [83]. The low binding affinity of BMP-2 to collagen leads to the uncontrolled release of collagen-encapsulated BMP-2 [84]. A novel and simple micropattern-based carrier of gelatin array BMP-2 [85] used a GelMA hydrogel micro membrane for the local and controlled release of BMP-2 [85–87]. BMP affects the cell differentiation of BMSCs and promotes bone regeneration by binding to the BMP receptor or heterodimer on the cell surface [88]. Studies have shown that low-dose alendronate sodium can inhibit osteoclast activity through the mevalonate pathway, enhance the proliferation and differentiation of osteoblasts, and induce human MSCs to undergo osteogenesis [89, 90]. In in vitro studies, fixing BMP-2, BMP-7 or alendronate onto the surface of a titanium alloy interbody fusion cage significantly improved the adhesion, proliferation and differentiation of BMSCs and had significant biological effects [91]. It has also been shown that the nanohydroxyapatite (n-HA)-resveratrol (RES)-chitosan (CS) micro complex can create a favorable microenvironment by local slow release and can be used as a multifunctional filler for the treatment of osteoporosis bone defects and fracture repair [92].

In the field of bone tissue engineering, significant progress has been made in the design of implants within the spine. Compared with traditional polymer materials, self-assembled supramolecular biomaterials have many advantages, including structural and functional tunability, contact reactivity, reversibility, and good biocompatibility. MSCs have been proven to effectively promote osteogenic differentiation [93]. However, due to factors such as manufacturing and industry supervision, scalability, immunogenicity, effectiveness, safety, the cost of grafts and structures, and other constraints, the clinical conversion rate of these therapies is very low [94]. Proteins are the executive molecules of all activities of in the body, and cell-derived exosomes/extracellular vesicles (EXOs/EVs) and synthetic liposomes have been widely used in heart disease, neurogenesis, osteochondral defects and tooth tissue repair [95, 96]. Exosomes are lipid bilayer-binding vesicles with a diameter of 50–150 nm and have specific surface markers [97–99]. Compared with stem cells in terms of tumorigenicity and immunogenicity, EXOs have better safety for use in vivo [100, 101]. However, clinically, due to their rapid metabolism, the administration of exosomes is limited to high-dose intravenous infusion or direct injection, the use period is short, and there is a risk of off-target effects [102]. Therefore, the development of a controlled release platform for complex biological agents has become the only way to promote and apply EXOs, ensuring that their integrity and biological activity are maintained during the encapsulation and release process. Polylactic acid-glycolic acid (PLGA) is widely used in the development of controlled release systems due to its controllable degradation rate and safe degradation byproducts [103]. Double embedding technology embeds proteins and small hydrophilic molecules into polymer microspheres [104]. Researchers designed a three-dimensional tissue engineering structure that combined PLLA nanofibers with porous implant scaffolds to guide bone regeneration by the local controlled release of stem cells, stem EXOs and EVs to reproduce the characteristics of MSCs with osteogenic advantages [105], providing infinite possibilities for the development and iterative optimization of new spine implant materials.

**Fig. 3:**
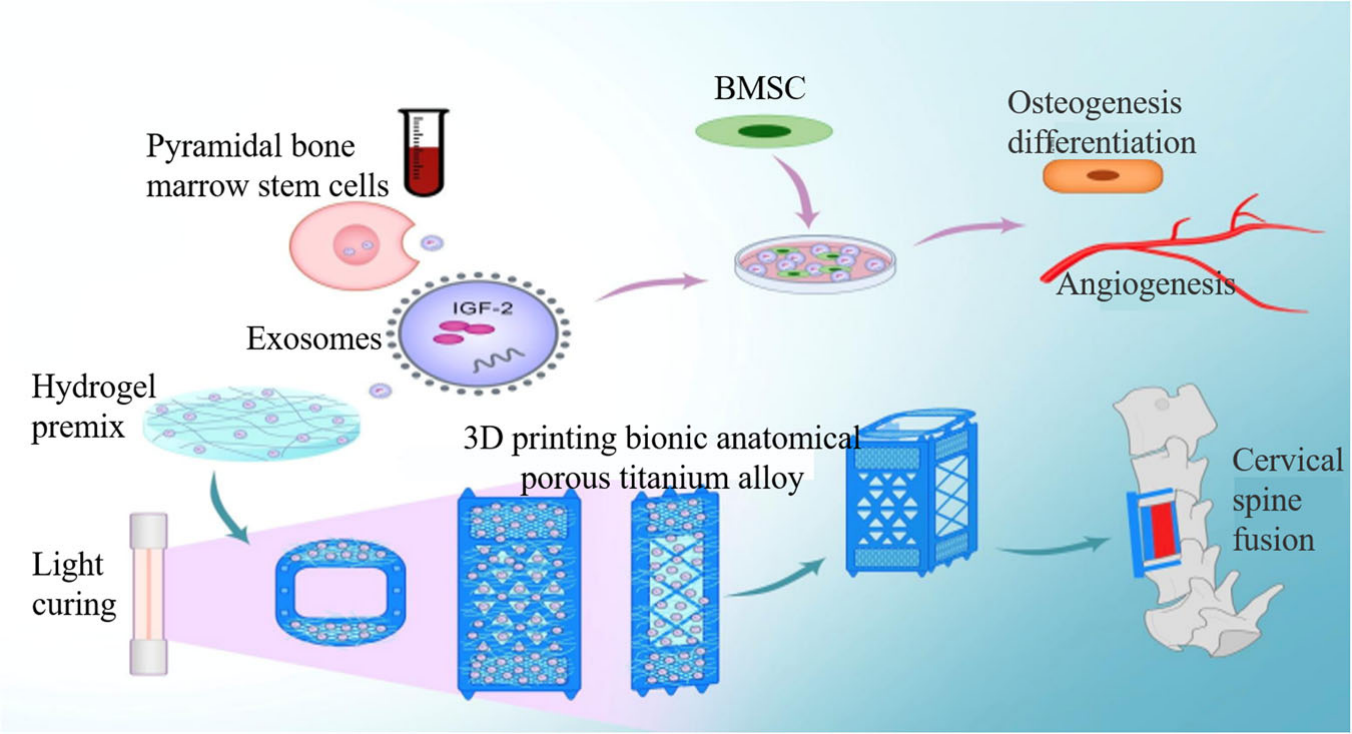
Vertebral bone marrow stem cell exosomes/biomimetic 3D printing porous titanium alloy scaffold construction and mechanism of promoting bone formation and angiogenesis in vivo and in vitro and promoting cervical fusion.

In the early stage, the research group used 3D bionic printed anatomical scaffold materials with the same endplate microstructure, premixed the obtained vertebral bone marrow stem cell exosomes into the hydrogel, and used light curing treatment and bionic 3D printing of porous titanium alloy scaffolds The interface of the endplate formed a tight adhesion, and finally an exosome-biomimetic 3D printed porous titanium alloy stent integrated graft was obtained (Fig. 3).The specific quality of bone fusion in the bone graft window and the growth of bone in the pore structure of porous titanium alloy stent after 3 months were evaluated by micro-CT and hard tissue section staining. The results showed that compared with the traditional titanium mesh, the new exosome-bionic 3D printed porous titanium alloy stent had better bone-metal interface integration and bone tissue growth in the pore structure.

### Four-dimensional (4D) bioprinting technology applied to spine materials

#### The development of 4D bioprinting technology

Fractures and surgical decompression of bone and joint degenerative diseases can lead to vertebral bone tissue defects, and it is usually necessary to use internal implants to promote bone regeneration and replace the defective tissue [106]. In the past two decades, as 3D bioprinting technology has made significant progress in bone tissue engineering, a large number of studies have combining biomaterials, cells and biologically active factors has become a development trend in the field of bone tissue engineering [107, 108]. With the continuous development of new material technologies, printing materials have also been developed from single solid powder materials such as metals, plastics, and ceramics mixed with materials such as liquids, gels, and cells [109]. These advanced technologies can promote the regeneration of bone tissue by using a controllable mode to construct bionic structure implants [110, 111]. As a kind of surgical implant product with clinical application prospects, the use of 3D-printing rapid prototyping technology is increasing and developing rapidly [112–114]. However, the clinical applications of 3D bioprinting in bone tissue engineering still face a series of challenges, such as tissue reconstruction of large and irregular bones and the personalized clinical needs of formed blood vessels and nerve regeneration [111, 115, 116]. 3D bioprinting can only print objects with static and inanimate initial conditions, while bone tissue regeneration includes a complex three-dimensional structure, microstructure and extracellular matrix composition, as well as the corresponding response to internal or external stimuli by endogenous mechanisms, which is finally accompanied by dynamic changes in tissue conformation from objects with unique functions. These dynamic functional conformation changes cannot be simulated by 3D bioprinting technology. Skylar Tibbits, the director of MIT’s Self-Assembly Laboratory, demonstrated four-dimensional (4D) printing technology for the first time in 2014. 4D bioprinting is a new generation of tissue engineering that integrates the concept of time as the fourth dimension into 3D bioprinting technology. This technology is expected to reshape the potential of implant product design in the spine. The two main features of 4D bioprinting are as follows: the shape or function of the biological endophyte, which is made based on the configuration of the printed object, does not change; and when external stimulation, cell fusion or self-assembly occurs after printing, the shape or function of the printed object also changes over time [117, 118]. The structure or function of the 4D-printed product is stable before and after stimulation, so the technology of 3D printing a structure with controllable degradation materials that completely disappears in the dynamic process will not be included in the scope of 4D printing [119]. Artificial induction or cell traction [120] can adjust the biological behavioral changes in cells and tissues, such as wound healing, angiogenesis, metastasis, and inflammation [120, 121] to achieve the structural deformation of endophytes while integrating 3D bioprinting technology to achieve 4D bioprinting [122–124].

#### The progress of 4D bioprinting technology

4D bioprinting of bone tissue implants can meet the needs of personalized bone regeneration by stimulating the shape change characteristics of materials [124]. Programmed cross-linking or recombination can regulate the mechanical properties of the internal implants [125], design self-folding microtubules [126] engineer vessels to achieve the spatiotemporal distribution and release of bioactive cells to promote the regeneration of bone, blood vessels and nerve tissues, and establish a long-term bionic microenvironment to enhance the osteogenic differentiation of stem cells [127]. With the deepening of 4D bioprinting technology research, scientific and technical guidance can be provided for the future clinical applications of spinal implants to manufacture fine-printed bone structures to dynamically adapt to bone defect areas [128–130].

#### Controlling factors of 4D bioprinting materials

4D bioprinted bone tissue implants can be stimulated by changes in the internal environment of the machine and temporal shape changes, so these changes can provide a tracing direction for the formulation of 4D bioprinted materials.

The extension and contraction of polymer chains caused by temperature changes are the most commonly used physical stimuli to achieve shape transformation. Therefore, a series of thermally responsive materials have been developed [131], such as poly-N-isopropylacrylamide [131, 132]. Changes in wettability and solubility with temperature cause biological materials to expand or contract and correspondingly deform [133–137]. Bioelectric stimulation can adjust the expansion, contraction or folding of endophyte biomaterials by changing the internal environment to affect the direction and intensity of the electric field, so hydrogels containing conductive polymers with good biocompatibility and printing performance should be selected for 4D bioprinting materials [138–141]. Magnetically responsive materials such as ferro- and paramagnetic nanoparticles (MNPs) can induce changes in the magnetic field force for controlled drug release [142, 143]. The combination of Fe_3_O_4_ nanoparticles and a polyethylene glycol/agar hydrogel network to construct a magnetically responsive drug delivery system provides a non-invasive treatment plan for clinical soft tissue injury treatment [143]. Magnetic hydrogels provide diverse choices for 4D bioprinting materials due to their good rheological properties [144, 145]. pH-responsive materials are generally biologically active factors attached to the surface of biological materials that contain chemical groups such as carboxyl, pyridine, sulfonic acid or phosphate that can release or accept protons with pH changes to cause the material to swell and change with pH changes [146].

#### The clinical application prospects of 4D bioprinting technology

A series of 4D bioprinting strategies allow science and technology to solve the problems of the in vivo transplantation of large-scale engineered bone graft substitutes and promote the regeneration of the microvascular system and neural network system [147–149]. Existing studies have combined mouse MSCs with methacrylate, alginate and hyaluronic acid mixed hydrogels and used 4D bioprinting technology to create hollow self-folding tubes that are active and equivalent in diameter to the smallest blood vessel [150]. 4D bioprinting technology that combines AM technology with the development of new materials and modern technology can directly print bioactive artificial bone tissue. The breakthrough of this technology is not far away. This technology can not only meet unresolved clinical medical needs but also completely change traditional material preparation methods. This emerging technology will provide enlightenment for the applications of spinal implant bone tissue engineering.

## Conclusions

This article reviews the effects of spinal implant materials on the biological response of bone tissue in vivo and in vitro. From the current research and literature review, it is obvious that simple changes in the physical properties of the surface of the endophyte material or changes in the chemical properties of the material can cause significant changes in the cellular response. With the advancement of science, the expectation is for continuous optimization and improvement in endophytes to promote future research and development in this field. Researchers have gradually developed methods that more directly couple biological reactions to the surfaces of metal materials. For this reason, the creation and development of 4D bioprinting technology that uses 3D printing technology to personalize customized-collective material surface modification technology-bioactive material coupling is expected to regenerate complex physiological functions after the implants are implanted into the body. A series of step-by-step 4D printing strategies have been proposed as new research and development directions for implants in the spine, but it is still challenging to print existing stimulus-responsive biomaterials and convert them into optimized bioinks, and further improvements in shape conversion are needed, including precise time-space controlled release and printing resolution. Undoubtedly, this new technology will provide a reliable scientific theoretical and technical basis for the research and clinical applications of biological functionalization of implants in the spine.

## References

[1] Levin JM, Tanenbaum JE, Steinmetz MP (2018). Posterolateral fusion (PLF) vs. transforaminal lumbar interbody fusion (TLIF) for spondylolisthesis: a systematic review and meta-analysis. Spine J.

[2] Gittens RA, Scheideler L, Rupp F, Hyzy SL, Geis-Gerstorfer J, Schwartz Z (2014). A review on the wettability of dental implant surfaces II: Biological and clinical aspects. Acta Biomater.

[3] Murphy Ciara M, Haugh Matthew G, O’Brien Fergal J (2010). The effect of mean pore size on cell attachment, proliferation and migration in collagen–glycosaminoglycan scaffolds for bone tissue engineering. Biomaterials..

[4] Khoda AKM, Ozbolat IT, Koc B (2013). Designing heterogeneous porous tissue scaffolds for additive manufacturing processes. Computer Aided Des..

[5] Wang Z, Hui A, Zhao H, Ye X, Zhang C (2020). A Novel 3D-bioprinted porous nano attapulgite scaffolds with good performance for bone regeneration. Int J Nanomed..

[6] Zhang M, Lin R, Wang X, Xue J, Wu X (2020). 3D printing of Haversian bone-mimicking scaffolds for multicellular delivery in bone regeneration. Sci Adv..

[7] Aldemir Dikici B, Reilly GC, Claeyssens F (2020). Boosting the Osteogenic and Angiogenic performance of multiscale porous polycaprolactone scaffolds by in vitro generated extracellular matrix decoration. ACS Appl Mater Interfaces..

[8] Zhen G, Wang R, Zhuo X, Li ZY, Yang XJ (2017). Incorporation of silver and strontium in hydroxyapatite coating on titanium surface for enhanced antibacterial and biological properties. Mater Sci Eng C Mater Biol Appl.

[9] Yamamoto M, Hokugo A, Takahashi Y, Nakano T, Hiraoka M, Tabata Y (2015). Combination of BMP-2-releasing gelatin/β-TCP sponges with autologous bone marrow for bone regeneration of X-ray-irradiated rabbit ulnar defects. Biomaterials.

[10] Yang G, Rothrauff BB, Lin H, Gottardi R, Alexander PG, Tuan RS (2013). Enhancement of tenogenic differentiation of human adipose stem cells by tendon-derived extracellular matrix. Biomaterials..

[11] Fernando GO, Ana RR, Geronimo P, Bráulio SA, Cristol PG, Joyce RA (2015). Understanding growth mechanisms and tribocorrosion behaviour of porous TiO2 anodic films containing calcium, phosphorous and magnesium. Appl Surf Sci..

[12] Lee SS, Hsu EL, Mendoza M, Ghodasra J, Nickoli MS, Ashtekar A (2015). Gel Scaffolds of BMP‐2‐Binding peptide amphiphile nanofibers for spinal arthrodesis. Adv Health Mater.

[13] Yao Q, Liu Y, Selvaratnam B, Koodali RT, Sun H (2018). Mesoporous silicate nanoparticles/3D nanofibrous scaffold-mediated dual-drug delivery for bone tissue engineering. J Control Release.

[14] Ceylan TD, Fabian F, Christian W, Andreas L (2018). A multifunctional multimaterial system for on-demand protein release. J Control Release.

[15] Oliveira HFD, Weiner AA, Majumder A, Shastri VP (2008). Non-covalent surface engineering of an alloplastic polymeric bone graft material for controlled protein release. J Controlled Release Off J Controlled Release Soc.

[16] Dumas A, Moreau MF, Ghérardi RK, Basl MF, Chappard D (2010). Bone grafts cultured with bone marrow stromal cells for the repair of critical bone defects: an experimental study in mice. J Biomed Mater Res A.

[17] Checchi M, Bertacchini J, Grisendi G, Smargiassi A, Palumbo C (2018). Proposal of a novel natural biomaterial, the scleral ossicle, for the development of vascularized bone tissue in vitro. Biomedicines.

[18] Ozbolat IT (2017). Bioprinting of osteochondral tissues: a perspective on current gaps and future trends. Int J Bioprint.

[19] Belka M, Ulenberg S (2017). Fused deposition modeling enables the low-cost fabrication of porous, customized-shape sorbents for small-molecule extraction. Anal Chem..

[20] Weng L, Boda SK, Wang H, Boda, Hong J, Wang (2018). Novel 3D hybrid nanofiber aerogels coupled with BMP2 peptides for cranial bone regeneration. Adv Health Mater.

[21] Jain S, Eltorai AEM, Ruttiman R, Daniels AH (2016). Advances in spinal interbody cages. Orthop Surg..

[22] Kuslich SD, Ulstrom CL, Griffith SL, Ahern JW, Dowdle JD (1998). The Bagby and Kuslich Method of lumbar interbody fusion: history, techniques, and 2-year follow-up results of a United States prospective, multicenter trial. Spine (Philos Pa 1976)..

[23] Kinaci A, Neuhaus V, Ring DC (2014). Trends in bone graft use in the United States. Orthopedics.

[24] Day SJ, Riley SP (2018). Utilising three-dimensional printing techniques when providing unique assistive devices: a case report. Prosthet Orthot Int..

[25] Kravitz Neal D, Groth C, Shannon T (2018). CAD/CAM software for three-dimensional printing. J Clin Orthod Jco..

[26] Baril E, Lefebvre LP, Hacking SA (2011). Direct visualization and quantification of bone growth into porous titanium implants using micro computed tomography. J Mater Sci Mater Med..

[27] Peng W, Xu L, You J, Fang L, Zhang Q (2016). Selective laser melting of titanium alloy enables osseointegration of porous multi-rooted implants in a rabbit model. Biomed Eng Online..

[28] Murphy CM, O’Brien FJ (2010). Understanding the effect of mean pore size on cell activity in collagen-glycosaminoglycan scaffolds. Cell Adh Migr.

[29] Charles S, Patrick T, Bruno J, Dumas M, Brailovski V (2017). Development of a porous metallic femoral stem: Design, manufacturing, simulation and mechanical testing. Mater Des..

[30] Xu N, Wei F, Liu X, Jiang L, Liang J, Liu ZJ (2016). Reconstruction of the upper cervical spine using a personalized 3D-printed vertebral body in an adolescent with Ewing Sarcoma. Spine (Phila Pa 1976).

[31] Yu D, Wang J, Qian KJ, Yu J, Zhu HY (2020). Effects of nanofibers on mesenchymal stem cells: environmental factors affecting cell adhesion and osteogenic differentiation and their mechanisms. J Zhejiang Univ Sci B..

[32] Bae I‐H, Yun K‐D, Kim H‐S, Jeong B-C, Lim H-P, Park S-W (2010). Anodic oxidized nanotubular titanium implants enhance bone morphogenetic protein-2 delivery. J Biomed Mater Res Part B Appl Biomater.

[33] Echeverry-Rendón M, Galvis O, Quintero Giraldo D, Pavón J, López-Lacomba JL, Jiménez-Piqué E (2015). Osseointegration improvement by plasma electrolytic oxidation of modified titanium alloys surfaces. J Mater Sci Mater Med.

[34] Karaji ZG, Hedayati R, Pouran B, Apachitei I, Zadpoor AA (2017). Effects of plasma electrolytic oxidation process on the mechanical properties of additively manufactured porous biomaterials. Mater Sci Eng C..

[35] Alves AC, Thibeaux R, Toptan F (2019). Influence of macroporosity on NIH/3T3 adhesion, proliferation, and osteogenic differentiation of MC3T3-E1 over bio-functionalized highly porous titanium implant material. J Biomed Mater Res B Appl Biomater.

[36] Harun WSW, Asri RIM, Alias J, Zulkifli FH, Kadirgama K, Ghani SAC (2018). A comprehensive review of hydroxyapatite-based coatings adhesion on metallic biomaterials. Ceramics Int.

[37] Ke D, Vu AA, Bandyopadhyay A, Bose S (2019). Compositionally graded doped hydroxyapatite coating on titanium using laser and plasma spray deposition for bone implants. Acta Biomater..

[38] Sarkar N, Bose S (2020). Controlled delivery of curcumin and vitamin K2 from hydroxyapatite-coated titanium implant for enhanced in vitro chemoprevention, osteogenesis, and in vivo osseointegration. ACS Appl Mater Interfaces.

[39] Surmenev RA, Surmeneva MA (2019). A critical review of decades of research on calcium phosphate–based coatings: How far are we from their widespread clinical application. Curr Opin. Biomed Eng.

[40] Porter AE, Patel N, Skepper JN, Best SM, Bonfield W (2003). Comparison of in vivo dissolution processes in hydroxyapatite and silicon-substituted hydroxyapatite bioceramics. Biomaterials.

[41] Ran J, Jiang P, Sun G, Ma Z, Hu J, Shen X (2017). Comparisons among Mg, Zn, Sr, and Si doped nano-hydroxyapatite/chitosan composites for load-bearing bone tissue engineering applications. Mater Chem Front..

[42] Norowski PA, Bumgardner JD (2010). Biomaterial and antibiotic strategies for peri-implantitis: a review. J Biomed Mater Res Part B Appl Biomater.

[43] Avés EP, Estévez GF, Sader MS, Sierra JCG, Yurell JCL, Bastoset IN (2009). Hydroxyapatite coating by sol–gel on Ti–6Al–4V alloy as drug carrier. J Mater Sci Mater Med.

[44] Antoci V, Adams CS, Parvizi J, Davidson HM, Composto RJ, Freeman TA (2008). The inhibition of Staphylococcus epidermidis biofilm formation by vancomycin-modified titanium alloy and implications for the treatment of periprosthetic infection. Biomaterials..

[45] Schmidmaier G, Lucke M, Wildemann B, Haas NP, Raschke M (2006). Prophylaxis and treatment of implant-related infections by antibiotic-coated implants: a review. Inj-Int J Care Injured..

[46] Zhu M, Liu X, Tan L, Cui Z, Liang Y, Li Z (2020). Photo-responsive chitosan/Ag/MoS2 for rapid bacteria-killing. J Hazard Mater.

[47] Bing C, Xiao F, Da X, Hu XL (2018). Polyprodrug antimicrobials: remarkable membrane damage and concurrent drug release to combat antibiotic resistance of methicillin‐resistant staphylococcus aureus. Small..

[48] Xiao Y, Peng J, Liu Q, Chen L, Qian Z (2020). Ultrasmall CuS-BSA nanoparticles with mild photothermal conversion. Theranostics..

[49] Huang D, Chen YS, Green CR, Rupenthal ID (2018). Hyaluronic acid coated albumin nanoparticles for targeted peptide delivery in the treatment of retinal ischaemia. Biomaterials..

[50] Kushwah V, Katiyar SS, Dora C (2018). Co-delivery of docetaxel and gemcitabine by anacardic acid modified self-assembled albumin nanoparticles for effective breast cancer management. Acta Biomater.

[51] Das RP, Singh BG, Kunwar A, Ramani MV, Subbaraju GV, Hassan PA (2017). Tuning the binding, release and cytotoxicity of hydrophobic drug by Bovine Serum Albumin nanoparticles: Influence of particle size. Colloids Surf B Biointerfaces.

[52] Geng Z, Ji L, Li Z, Wang J, He H, Cui Z (2021). Nano-needle strontium-substituted apatite coating enhances osteoporotic osseointegration through promoting osteogenesis and inhibiting osteoclastogenesis. Bioact Mater.

[53] Wang Z, Huang C, Wang J, Wang P, Shisheng BI, Asad Abbas CH. Design and simulation of flow field for bone tissue engineering scaffold based on triply periodic minimal surface. Singapore: Springer; 2019;32.

[54] Alghamdi HS, Bosco R, Vanden B, Jeroen JJP, Walboomers XF, Jansen JA (2013). Osteogenicity of titanium implants coated with calcium phosphate or collagen type-I in osteoporotic rats. Biomaterials..

[55] Zhang BJ, Li J, He L, Hao H, Jie W (2020). Bio-surface coated titanium scaffolds with cancellous bone-like biomimetic structure for enhanced bone tissue regeneration. Acta Biomater.

[56] Fina F, Goyanes A, Gaisford S, Basit AW (2017). Selective laser sintering (SLS) 3D printing of medicines. Int J Pharm.

[57] Hutmacher DW (2000). Scaffolds in tissue engineering bone and cartilage. Biomaterials.

[58] Lutolf MP, Hubbell JA (2005). Synthetic biomaterials as instructive extracellular microenvironments for morphogenesis in tissue engineering. Nat Biotechnol..

[59] Rezwan K, Chen QZ, Blaker JJ, Boccaccini AR (2006). Biodegradable and bioactive porous polymer/inorganic composite scaffolds for bone tissue engineering. Biomaterials..

[60] Miao S, Zhu W, Castro NJ, Nowicki M, Zhou X, Cui H (2016). 4D printing smart biomedical scaffolds with novel soybean oil epoxidized acrylate. Sci Rep..

[61] Gonzalez-Fernandez T, Rathan S, Hobbs C, Pitacco P, Freeman FE, Cunniffe GM (2019). Pore-forming bioinks to enable Spatio-temporally defined gene delivery in bioprinted tissues. J Control Release.

[62] Pötzinger Y, Rahnfeld L, Kralisch D, Fischer D (2019). Immobilization of plasmids in bacterial nanocellulose as gene activated matrix. Carbohydr Polym.

[63] Malcolm DW, Wang Y, Overby C, Newman M, Benoit DSW (2020). Delivery of RNAi-based therapeutics for bone regeneration. Curr Osteoporos Rep..

[64] Hu B, Yan L, Wang M, Zhu Y, Yong Z, Sui B (2018). Functional reconstruction of critical-sized load-bearing bone defects using a Sclerostin-targeting miR-210-3p-based construct to enhance osteogenic activity. Acta Biomater.

[65] Lu GD, Cheng P, Liu T, Wang Z (2020). BMSC-derived exosomal miR-29a promotes angiogenesis and osteogenesis. Front Cell Dev Biol..

[66] Tavernier G, Andries O, Demeester J, Sanders NN, Smedt SCD, Rejman J (2011). mRNA as gene therapeutic: how to control protein expression. J Control Release..

[67] Xie Q, Wang Z, Zhou H, Zhang Y, Fan X (2015). The role of miR-135-modified adipose-derived mesenchymal stem cells in bone regeneration. Biomaterials.

[68] Yang L, Li Y, Gong R, Gao M, Feng C, Liu T (2019). The Long Non-coding RNA-ORLNC1 regulates bone mass by directing mesenchymal stem cell fate. Mol Ther: J Am Soc. Gene Ther.

[69] Bi H, Wang D, Liu X, Wang G, Wu X (2020). Long non-coding RNA H19 promotes osteogenic differentiation of human bone marrow-derived mesenchymal stem cells by regulating microRNA-140-5p/SATB2 axis. J Bioences..

[70] Saravanan S, Vimalraj S, Lakshmanan G, Jindal A, Sundaramurthi D, Bhattacharya J. Chitosan-based biocomposite scaffolds and hydrogels for bone tissue regeneration[M]. 2019;7:413–42.

[71] Liu Z, Chang H, Hou Y, Wang Y, Zhou Z, Wang M (2018). Lentivirus-mediated microRNA-26a overexpression in bone mesenchymalstem cells facilitates bone regeneration in bone defects of calvaria in mice. Mol Med Rep..

[72] Zhao M, Qiao M, Oyajobi BO, Mundy GR, Chen D (2003). E3 ubiquitin ligase Smurf1 mediates core-binding factor alpha1/Runx2 degradation and plays a specific role in osteoblast differentiation. J Biol Chem.

[73] Xiong A, He Y, Gao L, Li G, Weng J, Kang B (2020). Smurf1-targeting miR-19b-3p-modified BMSCs combined PLLA composite scaffold to enhance osteogenic activity and treat critical-sized bone defects. Biomater Sci.

[74] Cidonio G, Cooke M, Glinka M, Dawson JI, Oreffo ROC (2019). Printing bone in a gel: using nanocomposite bioink to print functionalised bone scaffolds. Mater Today.

[75] Pati F, Song TH, Rijal G, Jang J, Kim SW, Cho DW (2015). Ornamenting 3D printed scaffolds with cell-laid extracellular matrix for bone tissue regeneration. Biomaterials..

[76] Yang JZ, Shrike Zhang Y, Yue K, Khademhosseini A (2017). Cell-laden hydrogels for osteochondral and cartilage tissue engineering. Acta Biomater..

[77] Lin YY, Schuphan J, Dickmeis C, Buhl EM, Commandeur U, Fischer H (2020). Attachment of ultralow amount of engineered plant viral nanoparticles to mesenchymal stem cells enhances osteogenesis and mineralization. Adv Health Mater.

[78] Nagahama K, Ueda Y, Ouchi T, Ohya Y (2007). Exhibition of soft and tenacious characteristics based on liquid crystal formation by introduction of cholesterol groups on biodegradable lactide copolymer. Biomacromolecules.

[79] Yang S, Huang Y, Jian P, Xie Z, Tu M (2020). Enhanced cell affinity and osteogenic differentiation of liquid crystal‐based substrate via surface bio‐functionalization. J Biomed Mat Res Part A.

[80] Soon CF, Omar WI, Berends RF, Nayan N, Basri H, Tee KS (2014). Biophysical characteristics of cells cultured on cholesteryl ester liquidcrystals. Micron.

[81] Dolanmaz D, Saglam M, Inan O, Dundar N, Alniacık G, Trak BG (2015). Monitoring bone morphogenetic protein-2 and -7, soluble receptor activator of nuclear factor-κB ligand and osteoprotegerin levels in the peri-implant sulcular fluid during the osseointegration of hydrophilic-modified sandblasted acid-etched and sandblasted acid-etched surface dental implants. J Periodontal Res.

[82] Rosanne MR, Irene MC, Simon S, Gang C, Brenton C, Georg F (2018). Delivery of the improved BMP-2-Advanced plasmid DNA within a gene-activated scaffold accelerates mesenchymal stem cell osteogenesis and critical size defect repair. J Control Release.

[83] Shi P, Chen K, Goh J (2013). Efficacy of BMP‐2 delivery from natural protein based polymeric particles. Adv Health Mater.

[84] Dimitriou R, Jones E, McGonagle D, Giannoudis PV (2011). Bone regeneration: current concepts and future directions. BMC Med..

[85] Ma C, Chang B, Jing Y, Kim H, Kim H, Liu XH (2018). Bio-inspired micropatterned platforms recapitulate 3D physiological morphologies of bone and dentinal cells. Adv Sci (Weinh, Baden-Wurtt, Ger).

[86] Kim JM, Kim WJ, Mi YK, Kim KP, Sang KK (2019). Development of hydrogel microparticle based RT-qPCR for advanced detection of BCR-ABL1 Transcripts. Biochip J..

[87] Yi MH, Lee JE, Kim CB, Lee KW, Lee KH (2020). Locally controlled diffusive release of bone morphogenetic protein-2 using micropatterned gelatin methacrylate hydrogel carriers. Biochip J.

[88] Trino LD, Bronze-Uhle ES, Ramachandran A, Lisboa-Filho PN, Mathew MT, George A (2018). Titanium surface bio-functionalization using osteogenic peptides: surface chemistry, biocompatibility, corrosion and tribocorrosion aspects. J Mech Behav Biomed Mater..

[89] Duque G, Rivas D (2010). Alendronate has an anabolic effect on bone through the differentiation of mesenchymal stem cells. J Bone Min Res..

[90] Mulcahy LE, Curtin CM, Mccoy RJ, O’Brien FJ, Duffy GP (2015). The effect of bisphosphonate treatment on the biochemical and cellular events during bone remodelling in response to microinjury stimulation. Eur Cell Mater.

[91] Kämmerer PW, Pabst AM, Dau M, Staedt H, Al‐Nawas B, Heller M (2020). Immobilization of BMP-2, BMP-7 and alendronic acid on titanium surfaces: Adhesion, proliferation and differentiation of bone marrow-derived stem cells. J Biomed Mater Res A..

[92] Li L, Yu M, Li Y, QL A, HYC, Meng ZA (2021). Synergistic anti-inflammatory and osteogenic n-HA/resveratrol/chitosan composite microspheres for osteoporotic bone regeneration. Bioact Mater..

[93] De Kok IJ, Peter SJ, Archambault M, Bos CVD, Cooper LF (2003). Investigation of allogeneic mesenchymal stem cell-based alveolar bone formation: preliminary findings. Clin Oral Implants Res..

[94] Heathman TRJ, Nienow AW, McCall MJ, Coopman K, Kara B, Hewitt CJ (2015). The translation of cell-based therapies: clinical landscape and manufacturing challenges. Regen Med..

[95] Sharma P, Mesci P, Carromeu C, Mcclatchy DR, Schiapparelli L, Iii JRY (2019). Exosomes regulate neurogenesis and circuit assembly. Proc Natl Acad Sci.

[96] Zhang S, Chuah SJ, Lai RC, Hui, James HP, Lim SK (2018). MSC exosomes mediate cartilage repair by enhancing proliferation, attenuating apoptosis and modulating immune reactivity. Biomaterials..

[97] Théry C, Witwer KW, Aikawa E, Alcaraz MJ, Anderson JD, Andriantsitohaina R, et al. Minimal information for studies of extracellular vesicles 2018 (MISEV2018): a position statement of the International Society for Extracellular Vesicles and update of the MISEV2014 guidelines. JEV. 2018;7:1. 10.1080/20013078.2018.1535750 PMC6322352 30637094

[98] Witwer KW, Bwm VB, Bruno S, Choo A, Dominici M, Gimona M (2019). Defining mesenchymal stromal cell (MSC)-derived small extracellular vesicles for therapeutic applications. J Extracell Vesicles..

[99] Swanson WB, Gong T, Zhang Z, Eberle M, Ma PX (2020). Controlled release of odontogenic exosomes from a biodegradable vehicle mediates dentinogenesis as a novel biomimetic pulp capping therapy - ScienceDirect. J Control Release.

[100] Ma ZJ, Yang JJ, Lu YB, Liu ZY, Wang XX (2020). Mesenchymal stem cell-derived exosomes: Toward cell-free therapeutic strategies in regenerative medicine. World J Stem Cells..

[101] Jafarinia M, Alsahebfosoul F, Salehi H, Eskandari N, Ganjalikhani-Hakemi M (2020). Mesenchymal stem cell-derived extracellular vesicles: a novel cell-free therapy. Immunol Invest..

[102] Lai CP, Mardini O, Ericsson M, Prabhakar S, Maguire CA, Chen JW (2014). Dynamic biodistribution of extracellular vesicles in vivo using a multimodal imaging reporter. ACS Nano.

[103] Danhier F, Ansorena E, Silva JM, Coco R, Breton AL, Préat V (2012). PLGA-based nanoparticles: an overview of biomedical applications. J Control Release.

[104] Wei G, Pettway GJ, Mccauley LK, Ma PX (2004). The release profiles and bioactivity of parathyroid hormone from poly(lactic-co-glycolic acid) microspheres. Biomaterials..

[105] Swanson WB, Zhang Z, Xiu K, Gong T, Ma PX (2020). Scaffolds with controlled release of pro-mineralization exosomes to promote craniofacial bone healing without cell transplantation. Acta Biomater..

[106] Daly AC, Freeman FE, Gonzalez-Fernandez T, Critchley SE, Nulty J, Kelly DJ (2017). 3D Bioprinting for Cartilage and Osteochondral Tissue Engineering. Adv Health Mater..

[107] Jones N (2012). Science in three dimensions: the print revolution. Nature.

[108] Amini AR, Laurencin CT, Nukavarapu SP (2012). Bone tissue engineering: recent advances and challenges. Crit Rev Biomed Eng.

[109] Rengier F, Mehndiratta A, Tengg-Kobligk HV, Zechmann CM, Unterhinninghofen R, Kauczor HU (2010). 3D printing based on imaging data: review of medical applications. Int J Computer Assist Radio Surg..

[110] Santos BL (2018). Spheroids of stem cells as endochondral templates for improved bone engineering. Front Biosci.

[111] Bose S, Roy M, Bandyopadhyay A (2012). Recent advances in bone tissue engineering scaffolds. Trends Biotechnol.

[112] Arslan-Yildiz A, El Assal R, Chen P, Guven S, Inci F, Demirci U (2016). Towards artificial tissue models: past, present, and future of 3D bioprinting. Biofabrication.

[113] Claire Y, Xuanyi M, Wei Z, Pengrui W, Kathleen LM, Jacob S (2019). Scanningless and continuous 3D bioprinting of human tissues with decellularized extracellular matrix. Biomaterials..

[114] Cui H, Nowick IM, Fisher JP, Zhang LG (2017). 3D Bioprinting for organ regeneration. Adv Health Mater..

[115] Ashammakhi N, Hasan A, Kaarela O, Byambaa B, Sheikhi A, Akhilesh K (2019). Advancing frontiers in bone bioprinting. Adv Health Mater..

[116] Qasim M, Dong SC, Lee NY (2019). Advancements and frontiers in nano-based 3D and 4D scaffolds for bone and cartilage tissue engineering. Int J Nanomed.

[117] Kong YL, Tamargo IA, Kim H (2014). 4D printing: multi-material shape change. Architectural Des..

[118] Gao B, Yang Q, Zhao X, Jin G, Ma Y, Xu F (2016). 4D Bioprinting for Biomedical Applications. Trends Biotechnol.

[119] Zhou Y, Huang WM, Kang SF, Wu XL, Lu HB, Fu J (2015). From 3D to 4D printing: approaches and typical applications. J Mech Sci Technol.

[120] Kraning-Rush CM, Califano JP, Reinhart-King CA, Laird EG (2012). Cellular traction stresses increase with increasing metastatic potential. PLoS One..

[121] Franze K (2013). The mechanical control of nervous system development. Development.

[122] Saravanan S, Vimalraj S, Thanikaivelan P, Banudevi S, Manivasagam G (2019). A review on injectable chitosan/beta glycerophosphate hydrogels for bone tissue regeneration. Int J Biol Macromol..

[123] Senatov FS, Niaza KV, Zadorozhnyy YM, Maksimkin AV, Kaloshkin SD, Estrin YZ (2016). Mechanical properties and shape memory effect of 3D-printed PLA-based porous scaffolds. J Mech Behav Biomed Mater.

[124] Zhang L, Yang G, Johnson BN, Jia X (2019). Three-dimensional (3D) printed scaffold and material selection for bone repair. Acta Biomater..

[125] Suo H, Zhang D, Yin J, Qian J, Wu ZL, Fu JZ (2018). Interpenetrating polymer network hydrogels composed of chitosan and photocrosslinkable gelatin with enhanced mechanical properties for tissue engineering. Mater Sci Eng C..

[126] Ashammakhi N, Ahadian S, Zengjie F, Suthiwanich K, Lorestani F, Orive G (2018). Advances and future perspectives in 4D bioprinting. Biotechnol J..

[127] Li C, Armstrong JP, Pence IJ, Kit-Anan W, Puetzer C, Carreira SC, et al. Glycosylated superparamagnetic nanoparticle gradients for osteochondral tissue engineering. Elsevier Sponsored Documents. 2018;176:24–33. 10.1016/j.biomaterials.2018.05.029 PMC6018621 29852377

[128] Kim HD, Amirthalingam S, Kim SL, Lee SS, Hwang NS Biomimetic materials and fabrication approaches for bone tissue engineering. Adv Health Mater. 2017;6. 10.1002/adhm.201700612 29171714

[129] Vaithilingam J, Sanjuan‐Alberte P, Campora S, Rance GA, Jiang L, Thorpe J (2019). Multifunctional bioinstructive 3D architectures to modulate cellular behavior. Adv Funct Mater.

[130] Zuyuan L, Siqi Z, Jijia P, Rui S, Hao L, Yalin L (2018). Time-responsive osteogenic niche of stem cells: a sequentially triggered, dual-peptide loaded, alginate hybrid system for promoting cell activity and osteo-differentiation. Biomater Guildf..

[131] Wang X, Sun Y, Peng C, Luo H, Wang R, Zhang D (2015). Transitional suspensions containing thermosensitive dispersant for three-dimensional printing. ACS Appl Mater Interfaces.

[132] Bakarich SE, Gorkin R, Panhuis MIH, Spinks GM (2015). 4D printing with mechanically robust, thermally actuating hydrogels. Macromol Rapid Commun.

[133] Peppas NA, Bures P, Leobandung W, Ichikawa HE (2000). Hydrogels in pharmaceutical formulations. Eur J Pharmaceutics Biopharmaceutics.

[134] Li L, Wang Q, Xu Y (2003). Thermoreversible association and gelation of methylcellulose in aqueous solutions. Nihon Reoroji Gakkaishi..

[135] Dai M, Picot OT, Verjans JMN, Haan LTD, Schenning APHJ, Peijs T (2013). Humidity-responsive bilayer actuators based on a liquid-crystalline polymer network. ACS Appl Mater Interfaces..

[136] De Haan LT, Verjans JMN, Bastiaansen, Cees WM, Schenning Albertus PHJL (2014). Humidity-responsive liquid crystalline polymer actuators with an asymmetry in the molecular trigger that bend, fold, and curl. J Am Chem Soc.

[137] Li YC, Zhang YS, Akpek A, Su RS, Khademhosseini A (2016). 4D bioprinting: The next-generation technology for biofabrication enabled by stimuli-responsive materials. Biofabrication..

[138] Okuzaki H, Kuwabara T, Funasaka K, Saido T (2013). Humidity‐sensitive polypyrrole films for electro‐active polymer actuators. Adv Funct Mater.

[139] Li S, Shu K, Zhao C, Caiyun W, Zaiping G, Gordon W (2014). One-step synthesis of graphene/polypyrrole nanofiber composites as cathode material for a biocompatible zinc/polymer battery. ACS Appl Mater Interfaces..

[140] Roshan DG, Jun LX (2018). Stimuli-Responsive Cationic Hydrogels in Drug Delivery Applications. Gels (Basel, Switz).

[141] Nazeer MA, Batool R, Kizilel S (2021). Stimuli-responsive drug delivery hydrogels. Soft Matter Biomed Appl.

[142] Filipcsei G, Csetneki I, Szilágyi A, Zrínyi M (2007). Magnetic field-responsive smart polymer composites. Oligomers Polym Compos Mol Imprinting.

[143] Ulbrich K, Holá K, Šubr V, Bakandritsos A, Tuček J, Zboril R (2016). Targeted drug delivery with polymers and magnetic nanoparticles: covalent and noncovalent approaches, release control, and clinical studies. Chem Rev.

[144] Zhang Q, Liu J, Yuan K, Zhengguo Z, Xiaowen Z, Xiaoming B (2017). A multi-controlled drug delivery system based on magnetic mesoporous Fe3O4 nanopaticles and a phase change material for cancer thermo-chemotherapy. Nanotechnology.

[145] Fenghua Z, Linlin W, Zhichao Z, Yanju L, Jinsong L (2019). Magnetic programming of 4D printed shape memory composite structures. Compos Part A Appl Sci Manuf.

[146] Nadgorny M, Xiao Z, Chen C, Connal LA (2016). 3D-printing of pH-responsive and functional polymers on an affordable desktop printer. ACS Appl Mater Interfaces..

[147] Palaveniene A, Tamburaci S, Kimna C, Glambaite K, Liesiene J (2018). Osteoconductive 3D porous composite scaffold from regenerated cellulose and cuttlebone-derived hydroxyapatite. J Biomater Appl.

[148] Barabaschi G, Manoharan V, Li Q, Bertassoni LE (2015). Engineering pre-vascularized scaffolds for bone regeneration. Adv Exp Med Biol..

[149] BPDS A, BG B, MFA C, MR B, EG B, SL B (2019). Development of a cell-free and growth factor-free hydrogel capable of inducing angiogenesis and innervation after subcutaneous implantation. Acta Biomater.

[150] Devillard CD, Mandon CA, Lambert SA, Blum LJ, Marquette CA (2018). Bioinspired multi-activities 4d printing objects: a new approach toward complex tissue engineering. Biotechnol J.

